# Enhanced Cathode‐Electrolyte Interphase for Prolonged Cycling Stability of Aluminum‐Selenium Batteries Using Locally Concentrated Ionic Liquid Electrolytes

**DOI:** 10.1002/anie.202500041

**Published:** 2025-02-25

**Authors:** Cheng Xu, Thomas Diemant, Shuting Zhang, Xu Liu, Stefano Passerini

**Affiliations:** ^1^ Helmholtz Institute Ulm (HIU) Electrochemical Energy Storage Helmholtzstraße 11 D-89081 Ulm Germany; ^2^ Karlsruhe Institute of Technology (KIT) P.O. Box 3640 D-76021 Karlsruhe Germany; ^3^ School of Energy and Environment & Z Energy Storage Center Southeast University 211189 Nanjing China; ^4^ Austrian Institute of Technology (AIT) Center for Transport Technologies Giefinggasse 4 1210 Vienna Austria

**Keywords:** Al−Se batteries, locally concentrated electrolytes, ionic liquids, solid electrolyte interphase, cathode/electrolyte interphase

## Abstract

Al−Se batteries (ASeBs) with high theoretical specific capacity and discharge voltage are promising energy storage devices. However, the detrimental shuttle effect occurring in conventional ionic liquid electrolytes (ILEs) challenges their development. Herein, a thicker cathode/electrolyte interphase (CEI) is constructed via employing locally concentrated IL electrolytes (LCILEs) to overcome these issues. It is demonstrated that LCILEs facilitate the incorporation of Emim^+^ into the electrode/electrolyte interphases, and, meanwhile, more Al−Cl species deposits are observed in the CEI. The formed CEI effectively prevents the dissolution of poly‐selenides, inhibiting their related parasitic reactions. These result in ASeBs, employing the LCILE, to deliver a specific discharge capacity of 218 mAh g^−1^ at 0.5 A g^−1^ after 100 cycles at 20 °C, while the cell using the neat ILE only maintains 38 mAh g^−1^ under the same conditions. Moreover, an Al−S cell operated in LCILEs reaches 578 mAh g^−1^ at 0.1 A g^−1^ after 150 cycles, which is also significantly better than 317 mAh g^−1^ in the neat ILE. This study provides an LCILE‐based strategy to reinforce the CEI in order to suppress the shuttle effect, realizing Al‐chalcogen batteries with better performance.

## Graphical abstract

The locally concentrated ionic liquid electrolyte prepared via diluting the ionic liquid electrolyte with non‐solvating 1‐chloro‐2‐fluorobenzene co‐solvent facilitates the deposition of Emim^+^ and Al−Cl species for the formation of a thicker cathode/electrolyte interphase (CEI) in Al−Se batteries. This prevents the dissolution of poly‐selenides and their deposition on Al metal anode, ultimately enhancing the cyclability of the batteries.

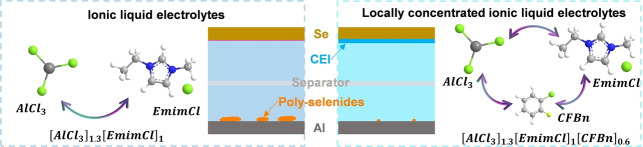

## Introduction

Al metal is recognized as one of the most powerful energy storage candidates due to its high theoretical capacity (2980 mAh g^−1^ and 8050 mAh cm^−3^), abundant reserves, low cost, and easy and convenient storage/transportation.[^1^, ^2^, ^3^, ^4^, ^5^] So far, non‐aqueous rechargeable Al‐metal batteries (AMBs) pairing Al metal anode and various cathode materials,[^6^, ^7^] e.g., carbon,[^8^, ^9^] oxides,^[10]^ sulfides,^[11]^ selenides,^[12]^ and organics have been reported.[^13^, ^14^] However, the issue of low specific capacity greatly limits the energy density improvement of AMBs.^[15]^

Based on this, chalcogen materials are promising cathode candidates for AMBs because of their high theoretical specific capacity,[^16^, ^17^] e.g., 1675 mAh g^−1^ for sulfur^[18]^ and 675 mAh g^−1^ for selenium.^[19]^ Compared with sulfur, selenium exhibits more intrinsic advantages, such as comparable theoretical volumetric capacity density (3247 mAh cm^−3^), higher ionic conductivity and output voltage, making it a compelling alternative despite its higher cost.^[20]^ Nonetheless, ASeBs also face the notorious shuttle effect and their related parasitic reactions leading to poor cyclic stability. To date, the most popular approach for addressing such obstacles is the manufacture of host materials.[^21^, ^22^, ^23^] For instance, Huang et al. developed a high surface area mesoporous carbon material as host for selenium nanowires to capture Se species achieving better cycling stability.^[24]^ Besides, electrolyte design and separator modification can be used to promote the electrochemical performance of ASeBs.[^25^, ^26^, ^27^] However, the electrolyte, an important component feasible to suppress the shuttle effect, has received little attention in ASeBs so far.

LCILEs, prepared by mixing non‐solvating, low‐viscosity co‐solvents with ILEs, exhibit lower viscosity and higher ionic conductivity with respect to the neat ILEs.[^28^, ^29^] Such an approach has been successfully applied for Li^+^,[^30^, ^31^, ^32^] Na^+^,^[33]^ K^+^,^[34]^ and Al^3+^ electrolytes, reducing the viscosity of highly concentrated electrolytes without affecting the local solvation of the active ionic species.[^35^, ^36^] Additionally, LCILEs have been demonstrated to be feasible in adjusting the electrode/electrolyte interphases (EEI) components for high‐performance Li−S batteries.^[37]^ These results indicate the possible application of LCILEs for ASeBs, although, to the best of our knowledge, constructing EEI to limit the shuttle effect in ASeBs through the LCILE strategy has not been reported.

Herin, a non‐ion‐solvating 1‐chloro‐2‐fluorobenzene (CFBn) as a model co‐solvent is firstly introduced into an ILE, i.e., [EmimCl]_1_[AlCl_3_]_1.3_ (EA), to form LCILEs (EACFBn) and its influence on ASeBs is further explored. As expected, CFBn promotes the fluidity and ionic conductivity without affecting the local ionic equilibrium. In addition, it is observed that CFBn also modifies both CEI and solid/electrolyte interphase (SEI) in ASeBs. Particularly, the modified CEI effectively prevents the dissolution of poly‐selenides and their related parasitic reactions, improving the Se species utilization. As a result, EACFBn‐based ASeB shows a specific discharge capacity of 218 mAh g^−1^ at 0.5 A g^−1^ after 100 cycles. In contrast, the capacity of an EA‐based cell is only 38 mAh g^−1^. In addition to the ASeB, an Al−S cell operated in EACFBn also achieves an improved cycling stability.

## Results and Discussion

**Figure 1:**
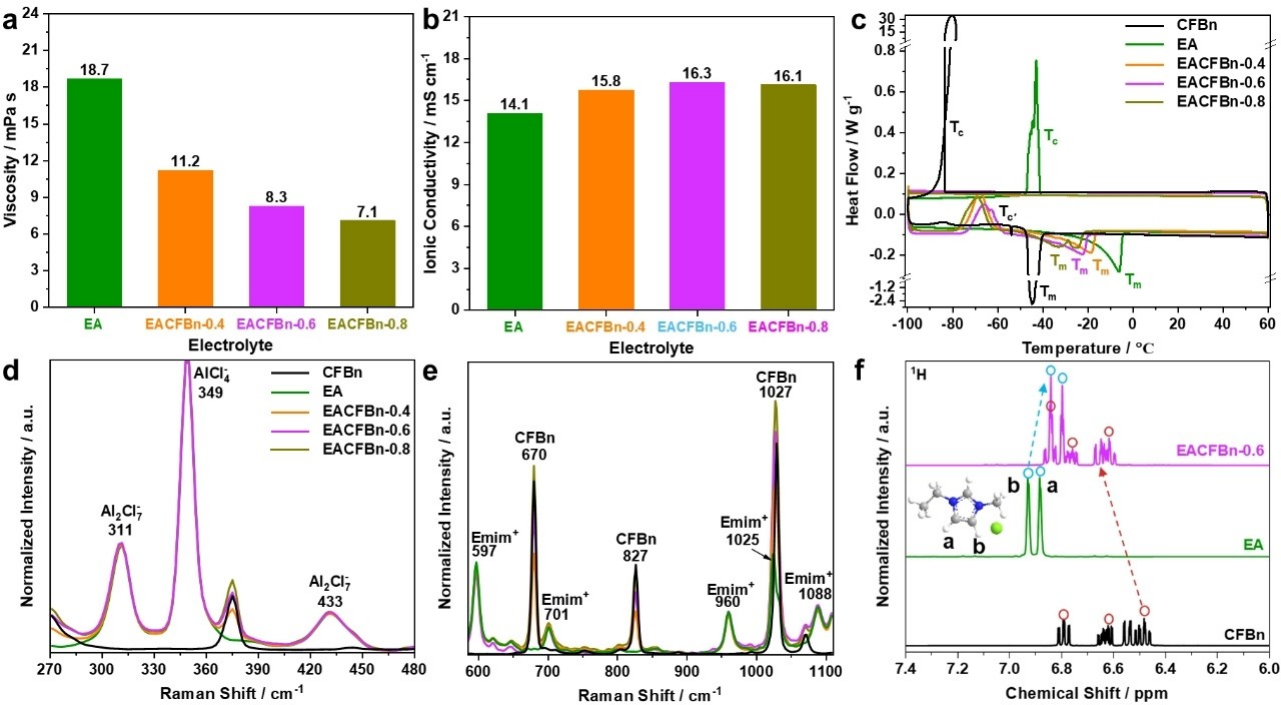
Physical properties of EA and EACFBn‐x electrolytes. (a) Viscosity at room temperature, (b) ionic conductivity at 20 °C. (c) DSC thermograms in the temperature range between −100 and 60 °C with a sweep rate of 5 °C min^−1^. Raman spectra in the ranges of (d) 270–480 and (e) 600–1100 cm^−1^. The Raman spectra of EA and EACFBn‐x electrolytes are normalized to the ${AlCl}_{4}^{-}$ peak at 349 cm^−1^. (f) ^1^H NMR spectra of CFBn, EA and EACFBn‐0.6.

The state‐of‐the‐art electrolyte for AMBs is the ILE [EmimCl]_1_[AlCl_3_]_1.3_. Adding a selected number of moles of CFBn co‐solvent, LCILEs, i.e., [EmimCl]_1_[AlCl_3_]_1.3_[CFBn]_x_, were obtained, referred as EACFBn‐x in the following. The comparison of the physical properties of EACFBn‐x versus neat EA is presented in Figure 1. As shown in Figure 1a and Table S1, the neat EA electrolyte displays a high viscosity of 18.7 mPa s caused by the large radius of the ionic species and their strong interactions. After the introduction of the co‐solvent CFBn, the viscosity decreases to 11.2 mPa s for EACFBn‐0.4, 8.3 mPa s for EACFBn‐0.6, and 7.1 mPa s for EACFBn‐0.8. This indicates that the addition of CFBn effectively improves the fluidity of ILEs. Figure 1b summarizes the ionic conductivity of the electrolytes. Among the LCILEs, EACFBn‐0.6 exhibits the highest ionic conductivity of 16.3 mS cm^−1^. As the CFBn molar content reached 0.8, the decrease of cationic and anionic charge carriers (Table S1) leads to a bell‐shaped ionic conductivity trend. In general, all LCILEs show an improved ionic conductivity compared with EA. This proves that CFBn facilitates the transport kinetics of ionic species. Next, the differential scanning calorimetry curves of the electrolytes in the temperature range between −100 °C and 60 °C are compared in Figure 1c. The results reveal that EA possesses a crystallization peak (*T*_c_) during the cooling scan and a melting peak (*T*_m_) during the heating scan, which are centered at −43 and −6 °C, respectively. By contrast, *T*_c_ disappears while a cold crystallization (T_c’_) appears for all EACFBn‐x, supporting for the formation of a glassy‐state at low temperatures. Also, *T*_m_ moves towards lower temperatures for these compounds. Altogether, EACFBn‐x possess wider liquidus ranges than neat EA.

The solvation structure of LCILEs was explored via Raman and ^1^H NMR spectroscopy. As illustrated in Figure 1d, the peaks of ${AlCl}_{4}^{-}$ (349 cm^−1^) and ${Al}_{2}{Cl}_{7}^{-}$ (311 and 433 cm^−1^) anions in the Raman spectra of EA and EACFBn‐x electrolytes show similar positions and normalized intensities. This means that the addition of CFBn does not affect the chemical equilibrium between ${AlCl}_{4}^{-}$ and ${Al}_{2}{Cl}_{7}^{-},$ which is crucial for efficient aluminum stripping/plating.^[38]^ The ${Emim}^{+}$ cation peaks are also not affected by the addition of CFBn as shown in Figure 1e. The ^1^H NMR spectra of CFBn, EA and EACFBn‐0.6 are compared in Figure 1f. The peaks at 6.8–7.0 and 6.4–7.0 ppm from EA and CFBn originate from the N‐CH=CH−N protons of the imidazolium ring and the protons of the benzene ring, respectively.^[35]^ In case of EACFBn‐0.6, the EA peaks show a lower chemical shift of 6.3–6.9 ppm. This change can be attributed to the charge transfer between ${Emim}^{+}$ and CFBn via π–π stacking.^[39]^

All these indicate that CFBn modifies the ${Emim}^{+}$ coordination environment but not the ${AlCl}_{4}^{-}$ and ${Al}_{2}{Cl}_{7}^{-}$ equilibrium. Also, the co‐solvent shows a good miscibility with EA, allowing the use of EACFBn‐x as electrolytes for Al‐metal batteries.

**Figure 2:**
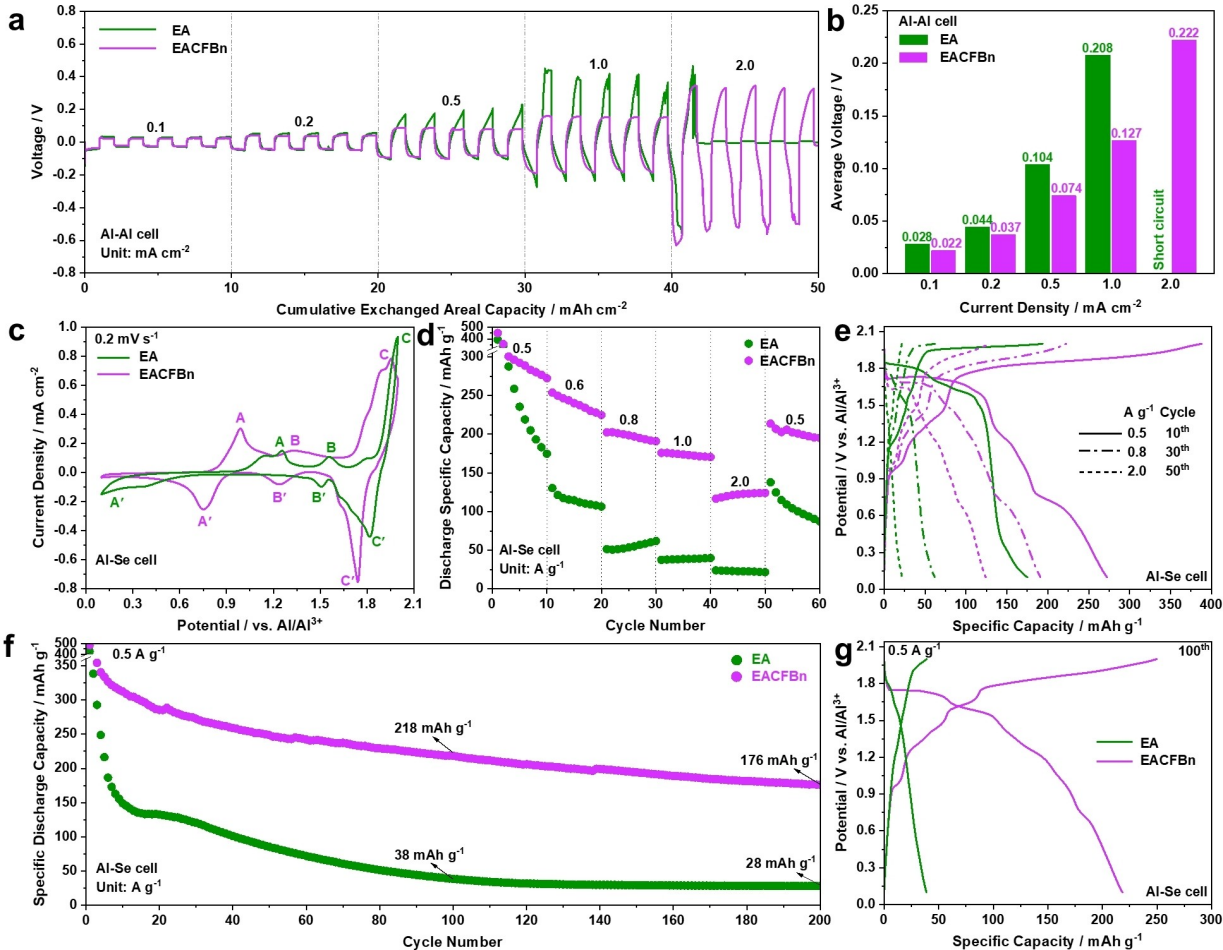
Electrochemical performance of Al−Al cells and ASeBs employing EA and EACFBn at 20 °C. (a) Rate capabilities of Al−Al cells with areal capacity of 1 mAh cm^−2^, and (b) corresponding average voltage at each current density. (c) CV curves of ASeBs with a scan rate of 0.2 mV s^−1^. (d) Rate capabilities of ASeBs, and (e) corresponding charge/discharge profiles. (f) Cycling performance of ASeBs at 0.5 A g^−1^, and (g) corresponding charge/discharge profiles of the 100^th^ cycle.

Next, the effect of CFBn on the electrochemical performance of the Al‐metal batteries is explored. Due to its highest ionic conductivity, EACFBn‐0.6 (named EACFBn in the following) was selected as the electrolyte of choice. In the first step, Al−Al symmetric cells employing EA and EACFBn were tested at current densities of 0.1, 0.2, 0.5, 1.0, and 2.0 mA cm^−2^ at 20 °C (Figure 2a). At lower current densities, 0.1 and 0.2 mA cm^−2^, both the EA‐ and EACFBn‐based Al−Al cells show aluminum stripping/plating profiles with an average voltage difference smaller than 100 mV (Figure 2b). However, the polarization of the EA‐based cell increases more rapidly at current densities equal or higher than 0.5 mA cm^−2^. At 1 mA cm^−2^, the average voltage difference between the two cells reaches 81 mV at 1 mA cm^−2^. Finally, the EA‐based cell experiences a short circuit at 2 mA cm^−2^, indicating for the occurrence of Al dendrites. Figure S1 compares the long‐term tests of Al−Al cells at 0.06 mA cm^−2^ with an areal capacity of 0.28 mAh cm^−2^, showing as the EACFBn‐based cell presents reversible aluminum stripping/plating behavior upon prolonged cycling. After cycling for 550 h, the EACFBn cell and an EA cell exhibit similar voltage profiles, confirming that it possesses good compatibility towards the Al anode.

In the next step, the compatibility of LCILEs towards chalcogen cathodes was explored, starting with the test of selenium‐based electrodes in LCILEs. ASeBs employing EA and EACFBn were assembled in three‐electrode T‐shaped cells consisting of a graphene@Se working electrode, an aluminum counter electrode, and an aluminum reference electrode. The graphene@Se composite has a Se content of 54 wt % analyzed by thermo‐gravimetric analysis (TGA, Figure S2). The cyclic voltammetry (CV) profiles of ASeBs in a voltage window of 0.1–2.0 V at a scan rate of 0.2 mV s^−1^ are summarized in Figure 2c. Three pairs of redox peaks are observed, corresponding to Se(−II)↔Se(0) (A−A’), Se(0)↔Se(I) (B−B’), and Se(I)↔Se(IV) (C−C’), respectively.[^19^, ^40^] Among these three redox processes, A−A’ shifts from 0.15–1.26 V for the EA‐based cell to 0.75–1.0 V for the EACFBn‐based cell, proving a significantly reduced activation barrier for the conversion of Se(−II) to Se(0) with EACFBn. In addition, B−B’ and C−C’ drop from 1.51–1.56 and 1.82–2.0 V to 1.24–1.32 and 1.74–1.95 V, respectively. Most importantly, the results illustrate that the use of EACFBn minimizes the polarization for the Se(−II) to Se(0) reaction significantly.

Figure 2d shows the rate capabilities of ASeBs with EA and EACFBn electrolyte at 20 °C. The EACFBn‐based cell displays reversible discharge specific capacities of 272, 225, 191, 171, 124, and 195 mAh g^−1^ after 10 cycles at 0.5, 0.6, 0.8, 1.0, 2.0, and 0.5 A g^−1^, respectively. In contrast, the EA‐based cell achieves 178, 106, 62, 40, 22, and 87 mAh g^−1^ under the same conditions. The comparison of voltage profiles shows that the EACFBn‐based cell possesses a lower charging plateau (Figure 2e and S3), indicating for a lower polarization. Figure 2f displays the performance of ASeBs during prolonged cycling at 0.5 A g^−1^. The EACFBn‐based cell shows a discharge specific capacity of 218 mAh g^−1^ after 100 cycles, which is significantly higher than the 38 mAh g^−1^ of EA‐based cell. After 200 cycles, the EACFBn‐based ASeB still maintains 176 mAh g^−1^ compared with the negligible capacity of 28 mAh g^−1^ in EA. Figure 2g and S4 summarize the evolution of the corresponding voltage profiles at various cycles. It can be seen that the capacity of the EA‐based ASeB decays rapidly within 10 cycles mainly due to the fast disappearance of the C’ region (i.e., the discharge capacity is reduced from 221 at the 2^nd^ cycle to 72 mAh g^−1^ at the 10^th^ cycle). These analyses demonstrate a high compatibility of EACFBn towards the Se cathode.

To understand the reason for the improved electrochemical performance, electrochemical impendence spectra (EIS) of ASeBs with EA and EACFBn electrolyte at the 1^st^ and 10^th^ cycle are compared in Figure S5. Clearly, these two electrolytes present a similar bulk resistance. However, a larger semi‐circle in the low‐frequency regions is observed in the EACFBn‐based cell, which becomes even larger after 10 cycles. Combined with the electrochemical performance of ASeBs, this finding underlines the influence of the electrolyte composition on the electrode/electrolyte interphases formation. Considering the increased impedance of the cathode, the reduced polarization observed in Figure 2c can be attributed to the improved kinetics of the Al metal anode (Figure 2a,b) and higher ionic transport in the electrolyte bulk (Figure 1b).

In order to investigate the surface morphology and chemical composition of the CEI on the cycled Se cathodes, scanning electron microscopy (SEM), energy dispersive X‐ray spectroscopy (EDX), and X‐ray photoelectron spectroscopy (XPS) were employed to characterize the electrodes after cycling in EA and EACFBn for 50 cycles. Before these measurements, the cycled electrodes were rinsed/cleaned by immersion in 1 mL dimethyl carbonate (DMC) for 2 min and then transferred into another fresh DMC batch for another 2 min, leading to 4 min in total to remove electrolyte's residues before characterization.

**Figure 3:**
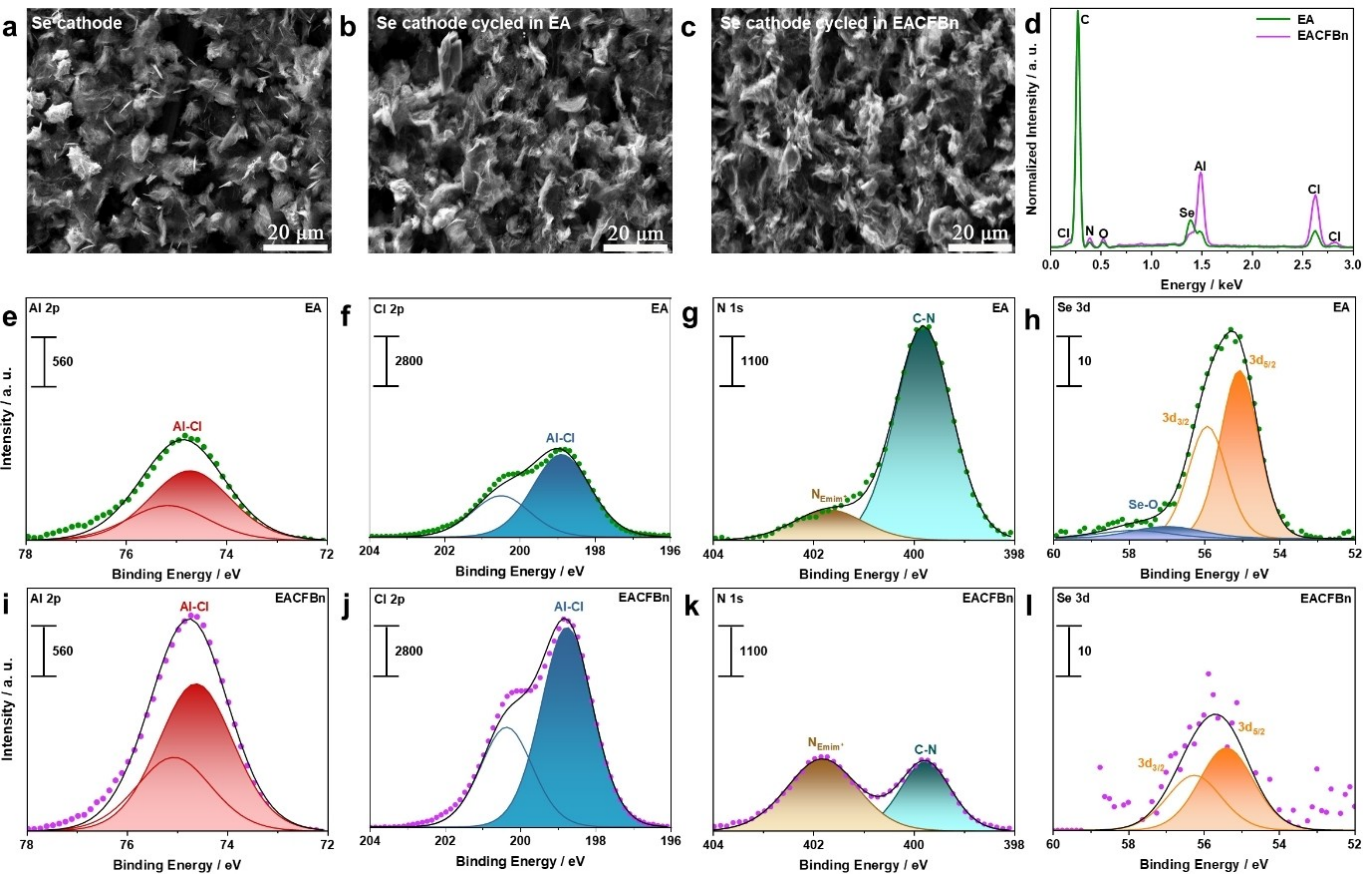
CEI characterization of the Se cathode after 50 cycles in EA and EACFBn. SEM image of (a) the initial Se cathode, Se cathodes cycled in (b) EA and (c) EACFBn. (d) EDX spectra of the cycled Se cathodes (intensity normalized to C peak). XPS detail spectra of the Se cathodes cycled in (e‐h) EA and (i‐l) EACFBn.

Figure 3a and S6 display the SEM morphology and elemental information of the initial Se cathode as determined by EDX mapping. In general, the porous morphology of the pristine sample is similar to the cycled Se cathodes as shown in Figure 3b and c. Figure 3d shows the normalized EDX spectra of Se electrodes after cycling (not normalized EDX spectra are shown in Figure S7). After cycling, stronger Se signals are observed in the EDX spectrum of the EA‐based electrode. Meanwhile, the Se cathode from the EACFBn‐based cell exhibits much higher concentration of Al and Cl signals. The comparatively weaker Se signal is probably caused by this enhanced deposition of Al and Cl species. To test the solubility of these Al and Cl deposits, the cycled Se cathode from an EACFBn‐based cell was subjected to DMC rinsing/cleaning with longer duration (8 min in total). As shown in Figure S8 and S9, the thoroughly cleaned Se cathode still shows stronger Al and Cl signals, indicating that much more insoluble Al−Cl species are deposited on the Se cathode surface in the EACFBn electrolyte.

In agreement to the EDX analysis (Figure 3d), the XPS results for the Se cathode cycled in EACFBn show a much higher concentration of Al and Cl species compared with EA (cf. Al 2p and Cl 2p detail spectra in Figure 3e, f, i, j). Two peaks at 401.9 and 399.9 eV are detected in the N 1s detail spectra (Figure 3g,k), which can be assigned to deposited $N_{{Emim}^{+}}$ and to the nitrile groups of the polyacrylonitrile (PAN) binder in the electrode material, respectively.^[41]^ Comparison of the intensities of the two contributions fits to the other results: the smaller intensity of the nitrile peak for the cathode cycled in EACFBn corroborates the formation of a thicker CEI. In contrast, $N_{{Emim}^{+}}$ presents a stronger signal after cycling in EACFBn, indicating a correlation between the presence of Emim^+^ cations and the CEI layer thickness. Furthermore, the Se cathode cycled in EACFBn exhibits also a lower intensity of Se 3d signal (Figure 3h, l), which is again in agreement with the formation of a thicker CEI layer on the surface of the Se cathode. Additionally, the in‐depth results of the electrode tested in EACFBn exhibit stronger Al 2p and Cl 2p signals, but weaker Se 3d signal with respect to the electrode tested in EA subjected to similar sputtering procedure with Ar^+^, effectively verifying the conclusion that a thicker CEI is generated in EACFBn (Figure S10). Surprisingly, the spectra in the F 1s region (Figure S11b,d) show not only for the sample cycled with EACFBn a fluorine signal but also for the EA sample. This could be due to the corrosiveness of Lewis‐acid ILs towards the PTFE cell body. Thus, the lower F signal area in EACFBn indicates that CFBn does not extensively contribute to the formation of the CEI. Taken together, these results prove that more Al, Cl, and Emim^+^ species contribute to the formation of the thickest CEI on the Se cathode surface after cycling in EACFBn, which may limit the dissolution of poly‐selenides.

**Figure 4:**
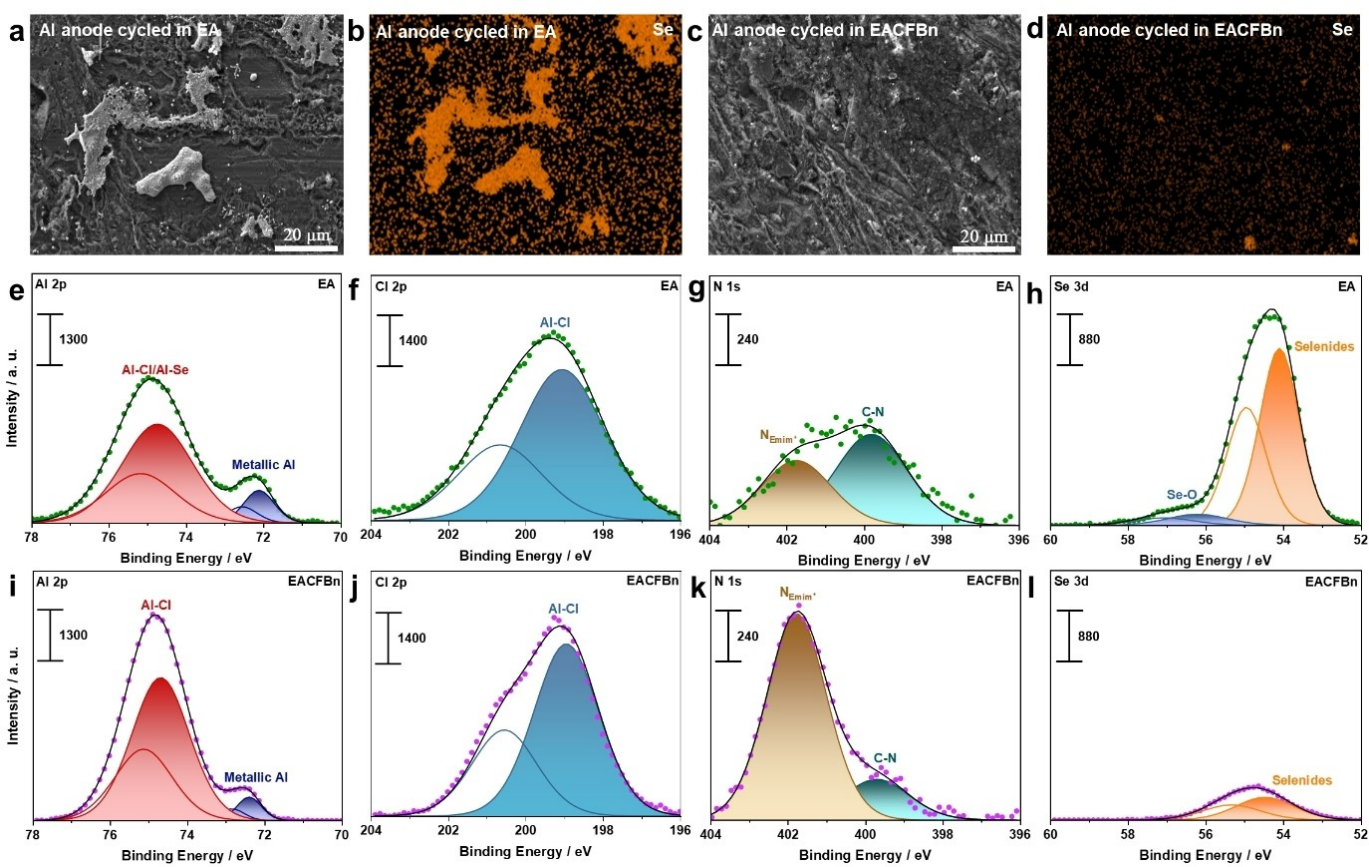
SEI characterization of the Al anode in EA and EACFBn after 50 cycles. SEM image of Al anodes cycled in (a) EA and (c) EACFBn. EDX mapping of Se signal in (b) EA and (d) EACFBn. XPS detail spectra of Al anodes cycled in (e–h) EA and (i‐l) EACFBn. Cycled anodes are cleaned by DMC before characterization.

In a further step, the surface morphology of the cycled Al anode in both electrolytes is discussed. Compared to the surface of an initial Al electrode (Figure S12), no obvious dendrite formation is observed after cycling in both electrolytes (Figure 4a, c). However, the Al anode cycled in EA clearly displays large‐sized and irregularly shaped deposits of Se species, as displayed in Figure 4b and S13a. This can be attributed to the shuttle of poly‐selenides from Se cathode to Al anode leading to parasitic reactions, in accordance with the fast deterioration of cycling stability in the EA‐based cells (cf. Figure 2d–g). In contrast, less Se deposition is observed after cycling with EACFBn (Figure 4d and S13b), suggesting that LCILE effectively helps to suppress the parasitic reaction at the Al anode side resulting in a good cycling performance.

The chemical information of the SEI species on the Al anodes cycled in EA and EACFBn are revealed using XPS (Figure 4e‐l and S14). The spectra in the Al 2p region (Figure 4e, i) show two peak doublets which can be assigned to metallic Al (Al 2p_3/2_ peak at 72.4 eV) and oxidized Al species (Al 2p_3/2_ peak at 74.7 eV) like Al−Cl compounds, respectively. Compared to EA, the Al anode cycled in EACFBn presents a weaker Al metal signal. Similar to the results at the cathode side, this finding suggests that a thicker SEI layer is formed on the Al anode surface upon cycling in EACFBn. In addition, the spectra in the N 1s region also show after cycling in EACFBn a stronger $N_{{Emim}^{+}}$ signal. Coming to the spectra in the Se 3d region (Figure 4h,l), the Al anode cycled in EA presents a much higher concentration of Se, which is a result of the more pronounced poly‐selenide shuttle to the Al anode side in this electrolyte.

Combining the findings from the XPS analysis (Figure 3 and 4), the physical properties of electrolytes (Figure 1), and the electrochemical performance of ASeBs (Figure 2), one can conclude that the addition of CFBn modifies the CEI through promoting the ionic transport of the electrolyte. The formation of CEI relies on ions reacting at the interface, necessitating ion transport from the electrolyte bulk to the surface of the electrode. CFBn effectively enhances the ionic transport ability of the electrolyte despite the reduced concentration of the ions (Figure 1b). Because of this, CFBn facilitates the formation of thicker CEI layers, including Emim^+^ and Al−Cl species, on the Se and Al surfaces. Particularly, the CEI prevents the dissolution of poly‐selenides into the electrolyte from the Se cathode side, and therefore limits the deposition of Se‐based species on the Al anode side, which result in better cycling performance of the cell. This phenomenon was also observed in our previous work regarding LCILEs for lithium metal batteries. The co‐solvent reduces the concentration of Emim^+^, but promotes its deposition on the surface of lithium metal anode.^[39]^ We also systemically investigate how the concentration of the non‐solvating co‐solvent not extensively participating in EEI formation affects the EEI formation.^[41]^

**Figure 5:**
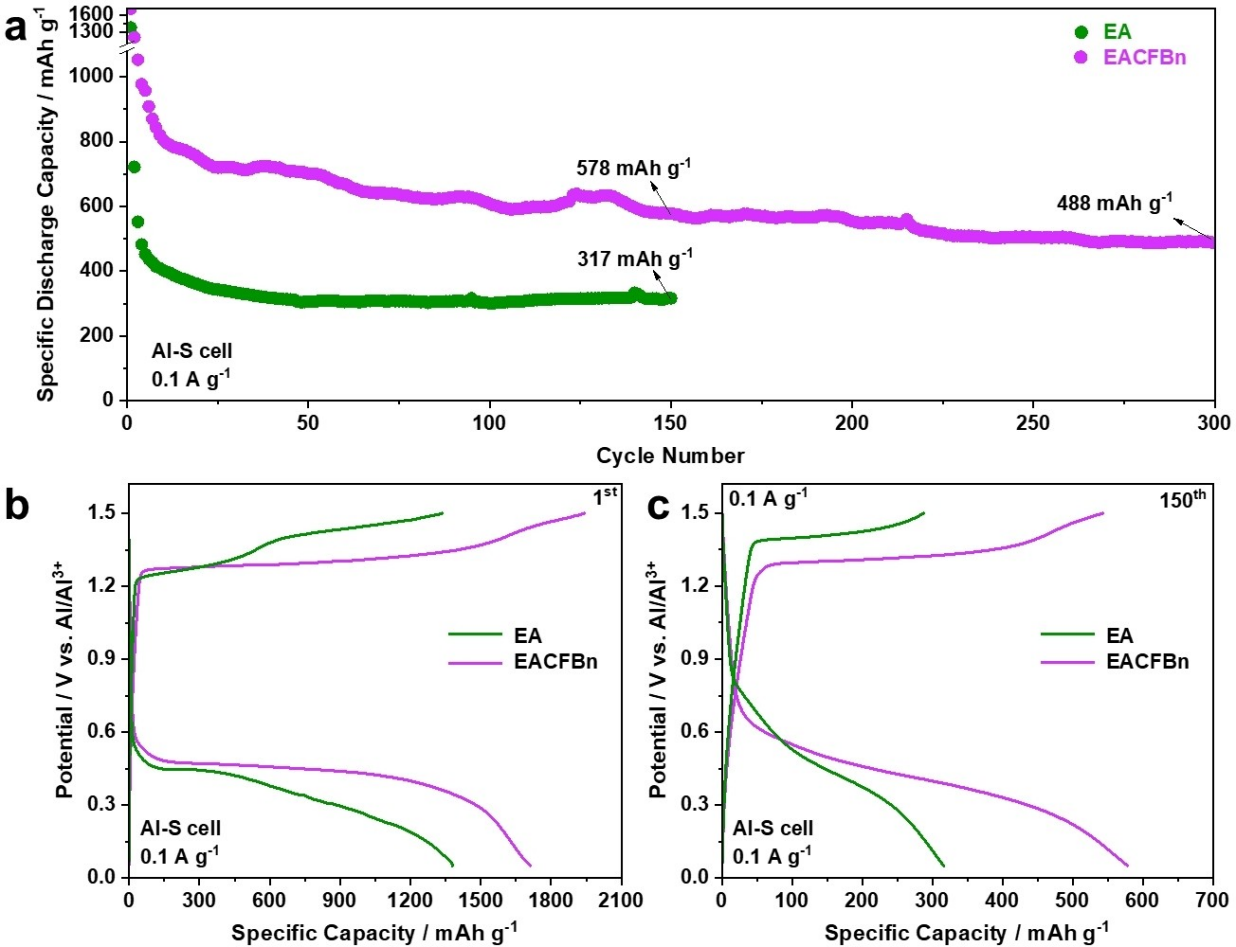
Electrochemical performance of Al−S cells employing EA and EACFBn at 20 °C. (a) Cycling performance at 0.1 A g^−1^, and corresponding dis‐/charge profiles of the (b) 1^st^ and (c) 150^th^ cycle.

In addition to ASeBs, the electrochemical performance of Al−S cells consisting of a sulfur cathode, an Al counter and an Al reference electrode in EA and EACFBn were also tested. Figure 5a presents the cycling performance with a current density of 0.1 A g^−1^ at 20 °C. The EACFBn‐based Al−S cell displays a discharge specific capacity of 578 mAh g^−1^ after cycling for 150 cycles, which is significantly higher than 317 mAh g^−1^ of EA, and still maintains 488 mAh g^−1^ after 300 cycles. In addition, the cell shows a lower polarization compared with that of EA (see Figure 5b,c). This result demonstrates that the LCILEs strategy also works on suppressing the shuttle effect of polysulfides for better‐performance Al−S cells. This optimized electrolyte can be combined with other strategies, such as the introduction of catalysts and separator modifications, to further reduce the cell polarization. For example, in one of our previous works,^[42]^ a three‐dimensional nitrogen‐doped carbonaceous networks anchored with cobalt was proposed as a separator modification layer, which effectively reduces the polarization of the sulfur cathode.

## Conclusion

LCILEs with the non‐solvating CFBn co‐solvent feature improved fluidity and ionic mobility compared to the neat EA electrolyte, and possess good compatibility towards Al anode and chalcogen cathodes. The results reveal that more Emim^+^ and Al−Cl species contribute to thicker CEI formation in LCILEs. The reinforced CEI prevents the dissolution of poly‐selenides into the electrolyte and limits their deposition on Al metal anode, hence boosting the utilization of Se. As a result, the modified ASeBs display a specific discharge capacity of 218 mAh g^−1^ at 0.5 A g^−1^ and 20 °C after 100 cycles in LCILEs. As a comparison, an EA‐based cell only retains 38 mAh g^−1^ under the same conditions. Moreover, an Al−S cell operated with EACFBn performs better, viz., attaining 578 mAh g^−1^ at 0.1 A g^−1^ after 150 cycles, compared to 317 mAh g^−1^ for EA. This work provides an LCILE strategy to improve the CEI and by that suppress the shuttle effect to achieve higher‐performance Al‐chalcogen batteries.

## Conflict of Interests

The authors declare no conflict of interest.

1

## Supporting information

As a service to our authors and readers, this journal provides supporting information supplied by the authors. Such materials are peer reviewed and may be re‐organized for online delivery, but are not copy‐edited or typeset. Technical support issues arising from supporting information (other than missing files) should be addressed to the authors.

Supporting Information

## Data Availability

The data that support the findings of this study are available on request from the corresponding author. The data are not publicly available due to privacy or ethical restrictions.
