# Supplementary material for: Enhanced Cathode‐Electrolyte Interphase for Prolonged Cycling Stability of Aluminum‐Selenium Batteries Using Locally Concentrated Ionic Liquid Electrolytes
Source: Angew Chem Int Ed Engl. 2025 Feb 25;64(17):e202500041. doi: 10.1002/anie.202500041 (PMC12015396; doi:10.1002/anie.202500041)
Supplement: Supplementary file 1 — Supporting Information [file ANIE-64-e202500041-s001.pdf]

Angewandte Chemie
Eine Zeitschrift der Gesellschaft Deutscher Chemiker

Supporting Information

Enhanced Cathode-Electrolyte Interphase for Prolonged Cycling Stability of Aluminum-Selenium Batteries Using Locally Concentrated Ionic Liquid Electrolytes

C. Xu, T. Diemant, S. Zhang, X. Liu, S. Passerini**

Supporting Information

Enhanced Cathode-Electrolyte Interphase for Prolonged Cycling Stability of Aluminum-Selenium Batteries Using Locally Concentrated Ionic Liquid Electrolytes

Cheng Xu, Thomas Diemant, Shuting Zhang, Xu Liu,* and Stefano Passerini*

Experimental Procedures

Synthesis of electrolytes. 1-Ethyl-3-methylimidazolium chloride (EmimCl, >98%, IoLiTec) was dried at 120 °C under vacuum for 20 h to remove residual water. Molecular sieves (3 Å, Alfa Aesar) were activated at 300 °C under vacuum for five days. EmimCl and molecular sieves were transferred into an argon-filled glovebox (< 0.1 ppm H_2O and O_2) after cooling down. All electrolytes were prepared in the glovebox. 1-Chloro-2-fluorobenzene (CFBn, VWR) was dried by the activated molecular sieves for more than three days before use. Anhydrous aluminum chloride ($AlCl_3$, ≥99.99, Alfa Aesar) was slowly added into EmimCl while stirring to reach a molar ratio of 1.3:1. The mixture was stirred at 130 °C for 30 mins and finally cooled down to room temperature (RT) and used in the following as EA electrolyte. The CFBn-containing electrolytes were synthesized via mixing EA electrolyte with CFBn at RT. The locally concentrated ionic liquid electrolytes (LCILEs) consisting of $AlCl_3$, EmimCl and CFBn in a molar ratio of 1.3:1:x (x = 0.4, 0.6, 0.8) were prepared and labelled as EACFBn-x.

Fabrication of electrodes. Chalcogen powders were prepared via a high-temperature melting method. 0.13 g graphene (The Sixth Element Inc.) and 0.23 g selenium powder (metals basis, Alfa Aesar) were mixed evenly and heated to 300 °C under vacuum (1×10^{-1} mbar) for 10 h. 0.11 g graphene and 0.35 g sulfur (99.98%, Sigma-Aldrich) were mixed evenly and encapsulated in a glass tube, and then heated to 155 °C in a vacuum of 1×10^{-1} mbar for 10 h. After cooling down and grinding, chalcogen powders with a selenium and sulfur content of 54 wt. % (graphene@Se) and 34 wt. % (graphene@S), respectively, were obtained. To prepare electrodes, N,N-dimethylformamide (DMF) solvent-based slurries consisting of the chalcogen powders, C65, and polyacrylonitrile (PAN, average M_w ~150000, Sigma-Aldrich) in a weight ratio of 70:15:15 were cast on non-graphitic carbon paper. After drying at 50 °C for 5 h, chalcogen disc electrodes with a diameter of 10 mm were obtained. The average mass loading of selenium and sulfur for graphene@Se and graphene@S electrodes were 1 and 0.5 mg cm^{-2}, respectively, corresponding to 0.68 and 0.84 mAh cm^{-2} capacity.

Electrochemical measurements. Al-chalcogen batteries were assembled inside a glovebox in three-electrode T-shaped cells (PTFE), employing a chalcogen working electrode, and Al foil counter and reference electrodes. 120 μL of the electrolyte were injected in each cell. Modified GF/D sheets

were used as separator, where the side facing the chalcogen electrodes was covered by a suspension of multi-walled carbon nanotubes (MWCNT, O.D. × L 6-9 nm × 5 μm, Sigma-Aldrich) with PAN as binder and DMF as solvent. The mass loading of MWCNT on the modified separators was ~0.2 mg cm^{-2}. Aluminum metal (0.1mm, 99.99%, Thermo) was cut to discs with a diameter of 10 mm, which were polished by sand paper in the glovebox before use. Furthermore, Al/Al symmetric cells were assembled with polished Al electrodes and a bare GF/D separator. All cells were tested with an external MACCOR series 4000 battery cycler via connecting to glass carbon electrode poles to investigate the electrochemical performance.

Material characterization. The measurements to determine the density and viscosity of electrolytes were carried out in the dry room (dew point < -60 °C). The density of the electrolytes was determined with the Density Meter DMA 4100 M (Anton Paar). The viscosity of the electrolytes was measured by a capillary viscosimeter and averaged over three measurements (**Table S1**). High-temperature conductivity cells (HTCC, Material Mates) with Pt-black electrodes were employed to study the ionic conductivities. More precisely, 0.5 mL electrolyte were filled in HTCCs and tested via electrochemical impedance spectroscopy by an integrated liquid conductivity system MCS 10 (Material Mates-Biologic). The average ionic conductivities were calculated from 60 data points. For each measurement, the HTCC constant was determined using 0.5 mL of the 0.01 m KCl standard solution. Differential scanning calorimetry (DSC) measurements were carried out with a differential scanning calorimeter (TA Instruments Q2000) under liquid N_2 cooling. The investigated electrolytes were sealed in aluminum pans inside the glove box and tested in the temperature range between -100 °C and 60 °C with a scanning rate of 5 °C min^{-1}. Thermo-gravimetric analysis (TGA) was carried out with a heating rate of 10 °C min^{-1} between 30 and 600 °C under N_2 atmosphere. ^1H NMR spectra of electrolytes were measured using a JNMR-GX 400 at 400 MHz with deuterium oxide (D_2O) as lock and chemical shift reference. NMR spectra were analyzed by MestReNova. Raman spectra of the electrolytes were obtained by a RAM II FT-Raman module of a Bruker Vertex70v FT-IR spectrometer with a laser wavelength of 1064 nm. Cyclic voltammetry (CV) and electrochemical impedance spectroscopy (EIS) data were collected via a galvanostat/potentiostat VMP2 (Bio-Logic, France). Cycled electrodes extracted from Al-chalcogen and Al/Al cells were soaked in DMC twice for 4 or 8

min and dried under vacuum at RT in glovebox. The morphology of active materials and electrodes was investigated via scanning electronic microscopy (SEM, Zeiss LEO 1550 microscope equipped with a EDX detector). X-ray photoelectron spectroscopy (XPS) measurements were carried out on a Specs XPS system with a Phoibos 150 energy analyzer using monochromatic Al-Kα radiation (1486.6 eV) and a pass energy of 30 eV at the analyzer for the detail spectra. The XPS data were analyzed by Casa XPS software and all spectra were calibrated to the main C 1s peak (of C-C/C-H species) at 284.8 eV.

Table S1. Flow times, density, molarity of ions (including cations and anions) and viscosity of EA and EACFBn.

	Flow time / s	Average flow time (t) / s	Density (ρ) / g mL^{-1}	Molarity of ions (M_v) / mol L^{-1}	Dynamic viscosity (μ) / mPa s
EA	279.13<br>282.21<br>280.43	280.59	1.336	8.248	18.68
EAdF-0.4	171.69<br>169.92<br>170.13	170.58	1.320	7.018	11.22
EAdF-0.6	126.35<br>125.87<br>128.75	126.99	1.310	6.512	8.29
EAdF-0.8	111.23<br>110.08<br>109.14	110.15	1.299	6.064	7.13

Average flow times were determined in three parallel experiments.

The dynamic viscosity μ was obtained by the following equation:

$$\mu = v \cdot \rho = k \cdot t \cdot \rho$$

where v, k, and t are the kinematic viscosity, instrument constant (0.04983 mm^2 s^{-2}), and average flow time, respectively.

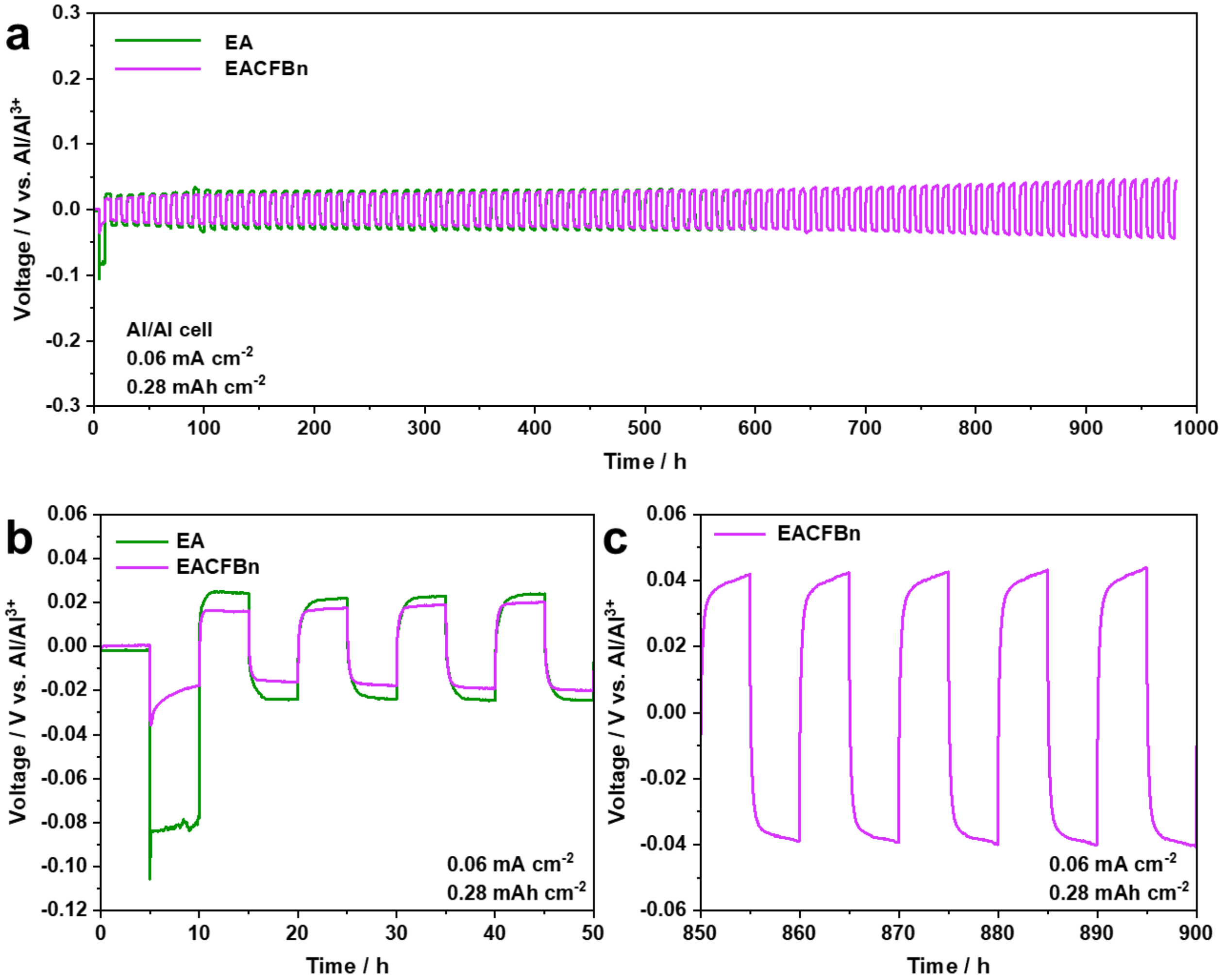

Figure S1. (a) Voltage profiles of Al/Al cells upon prolonged cycling at 0.06 mA cm^{-2} with an areal capacity of 0.28 mAh cm^{-2} per cycle. Voltage profiles during (b) initial 50 h and (c) 850-900 h.

The average voltage increases from 21 mV at the 10th cycle (100-105 h) to the 28 mV at the 60th cycle (600-605) as shown in Figure S1a, supporting for a decent cyclability of the metal anode in this electrolyte. Particularly, the performance of Al-Se batteries is currently mainly limited by the dissolution of poly-selenide from the cathode side, as discussed in the manuscript.

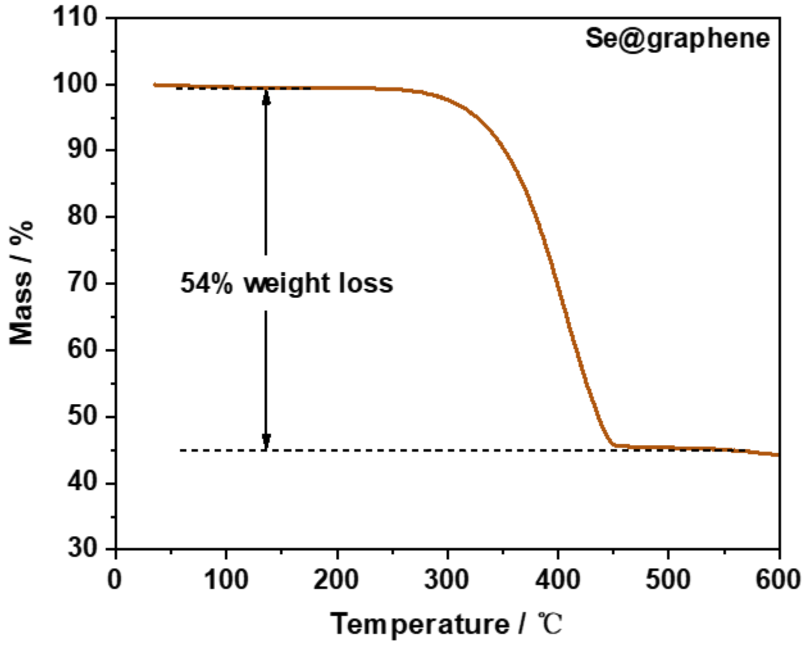

Figure S2. TGA of graphene@Se powder.

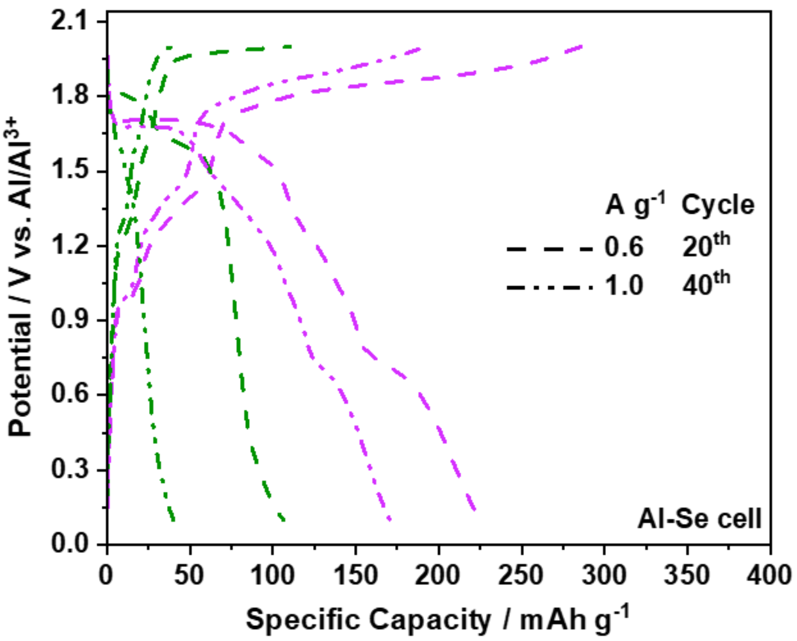

Figure S3. Charge/discharge profiles of Al-Se cells for the 20th (at 0.6 A g^{-1}) and 40th cycle (at 1.0 A g^{-1}).

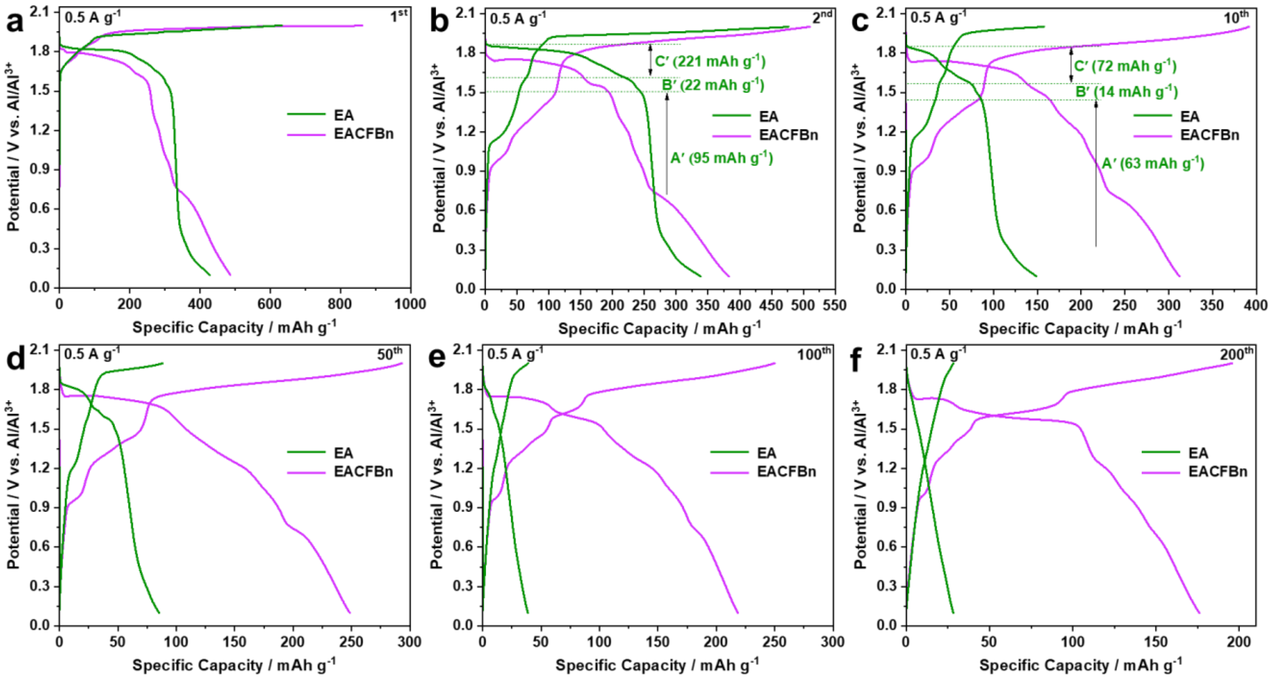

Figure S4. Charge/discharge profiles of Al-Se cells for varying cycles at 0.5 A g^{-1}.

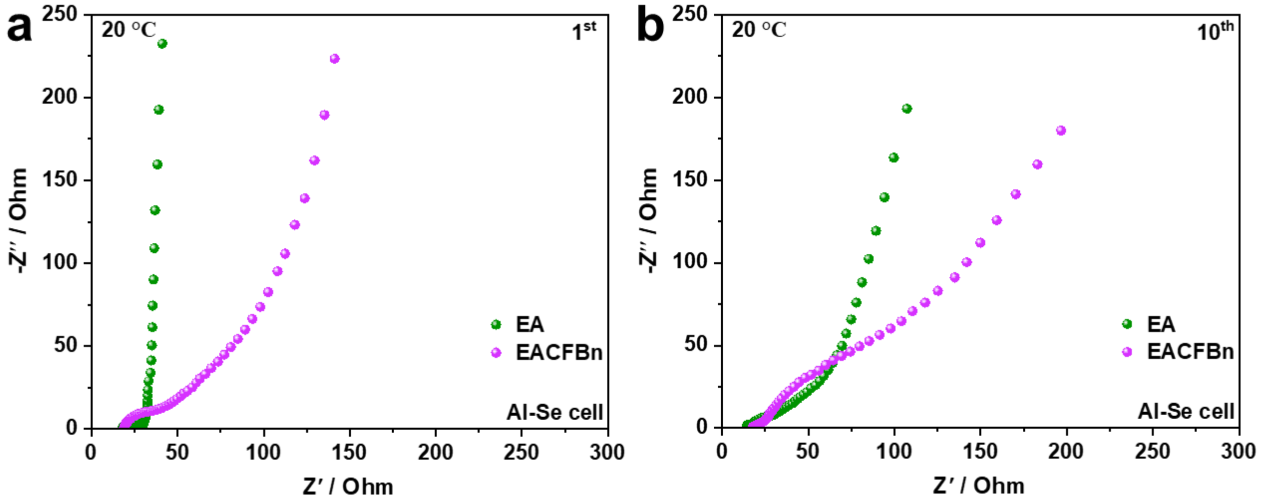

Figure S5. Nyquist plots of Al-Se cells with EA and EACFBn electrolyte at the (a) 1st and (b) 10th cycle.

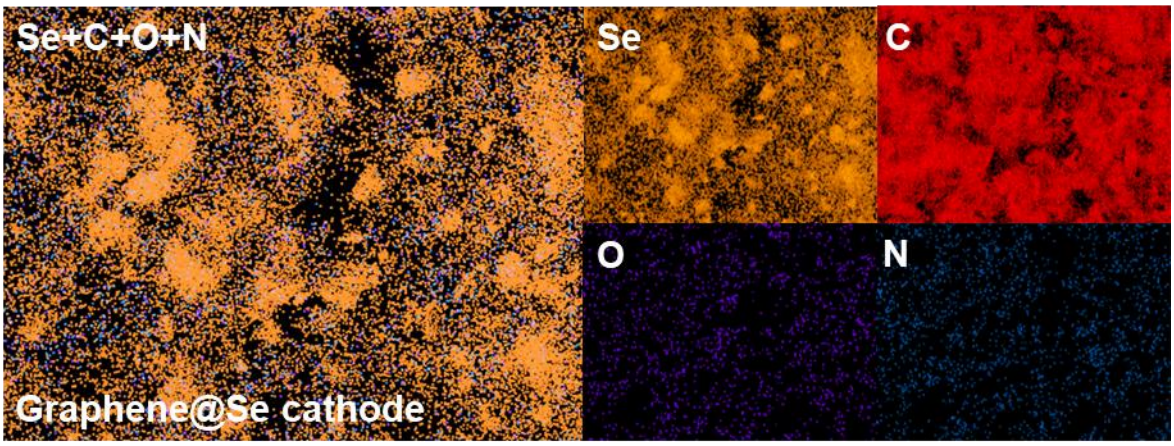

Figure S6. EDX mapping of the initial Se cathode.

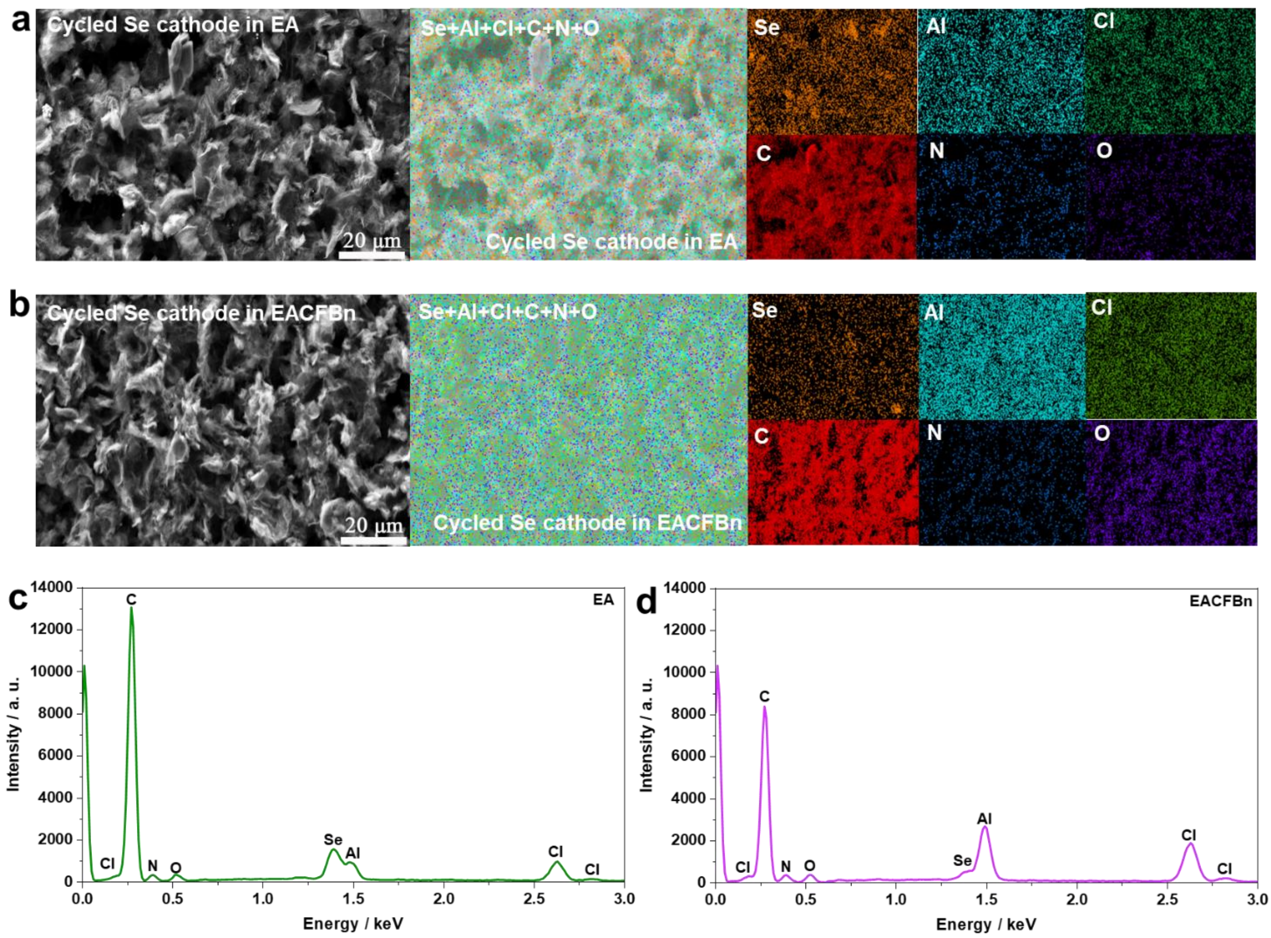

Figure S7. SEM image and corresponding EDX mapping of Se cathodes cycled in (a) EA and (b) EACFBn. EDX spectra (as-received original data) of the cycled Se cathodes cycled in (c) EA and (d) EACFBn.

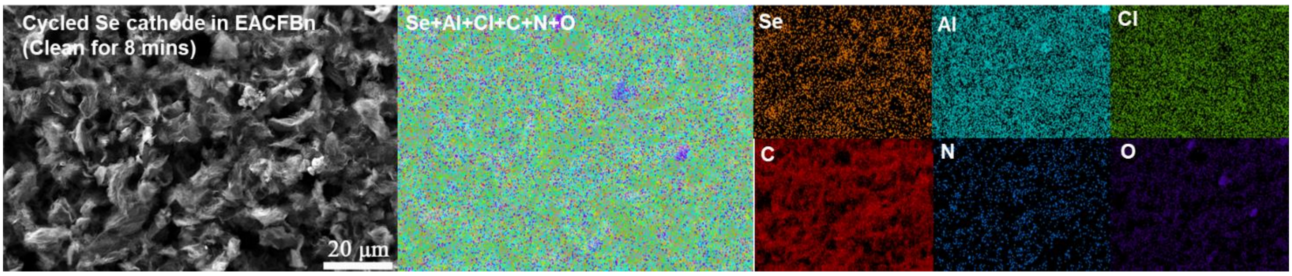

Figure S8. SEM image and EDX mapping of Se cathode cycled in EACFBn. The cathode was rinsed with DMC four times (each time for 2 min, 8 min in total).

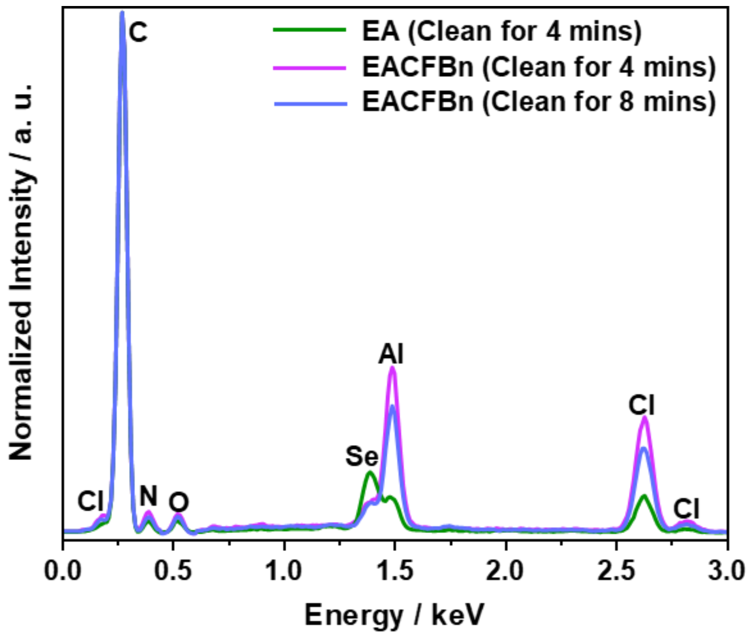

Figure S9. EDX spectra of Se cathodes cycled in EA and EACFBn (intensity normalized to C peak). The cathode cycled in EACFBn was rinsed with DMC four times (each time for 2 min, 8 min in total).

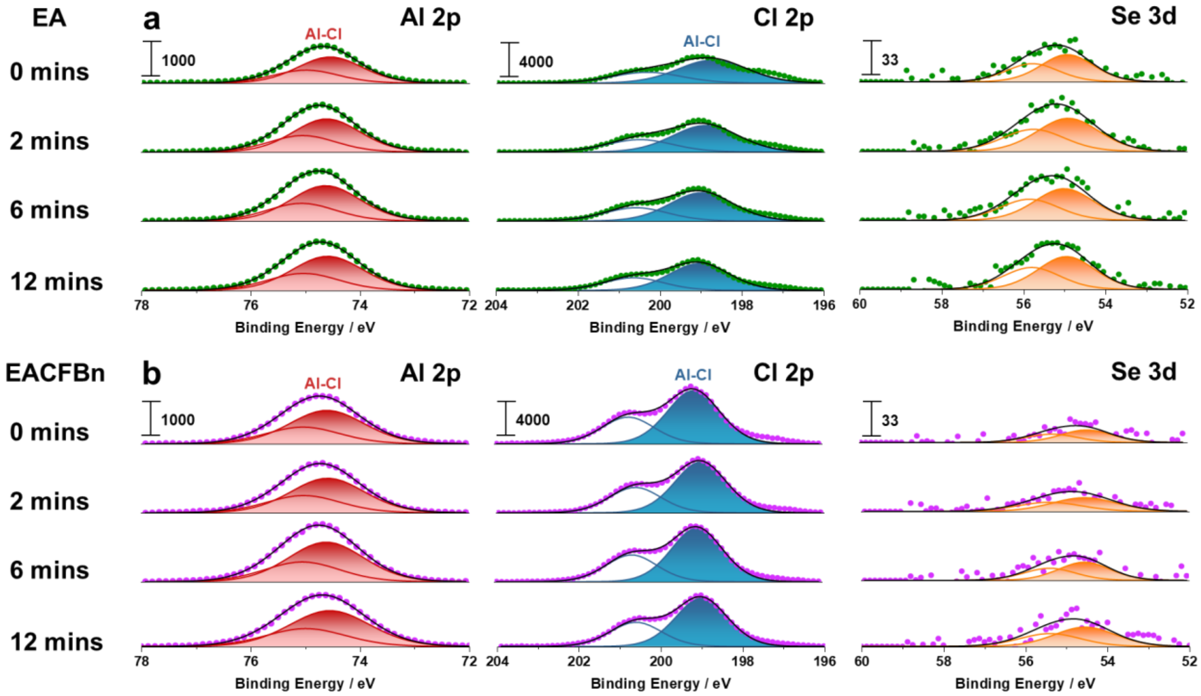

Figure S10. In-depth XPS spectra of cycled Se cathodes in (a) EA and (b) EACFBn after Ar^+ sputtering for different time.

Upon Ar^+ sputtering, the signal of Se 3d increases, indicating that the concentration of Se species increases. Also, despite a reduced intensity, the Al 2p and Cl 2p signals are still observed after sputtering for 12 min, indicating that CEI's species deeply extend into the electrode. This can be attributed to a highly porous morphology of the cathodes, as observed in Fig. 3b,c. Additionally, it is observed that the electrode tested in EACFBn exhibits stronger signal in Al 2p and Cl 2p, but weaker signal in Se 3d, with respect to the electrode tested in EA, verifying the conclusion that a thicker CEI is generated in EACFBn.

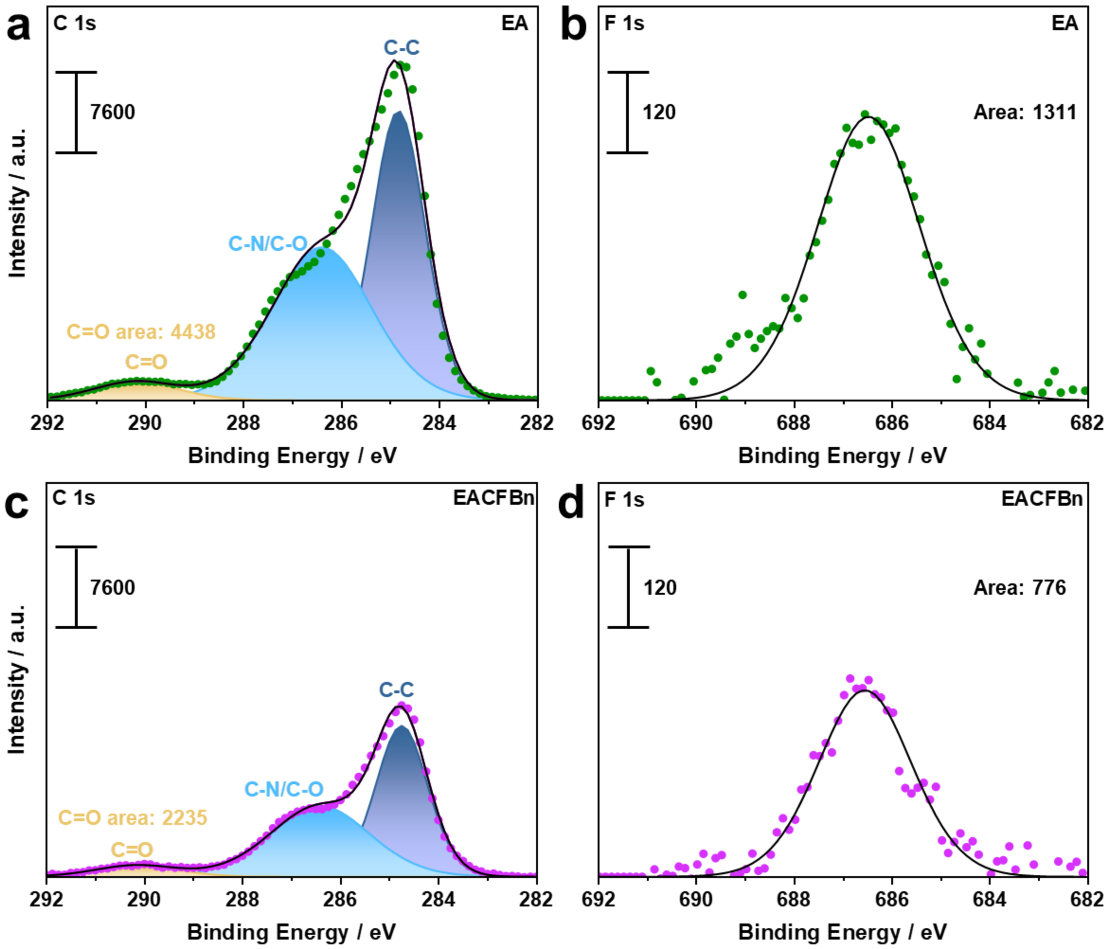

Figure S11. XPS detail spectra in the C 1s and F 1s regions of Se cathodes cycled in (a,c) EA and (b,d) EACFBn.

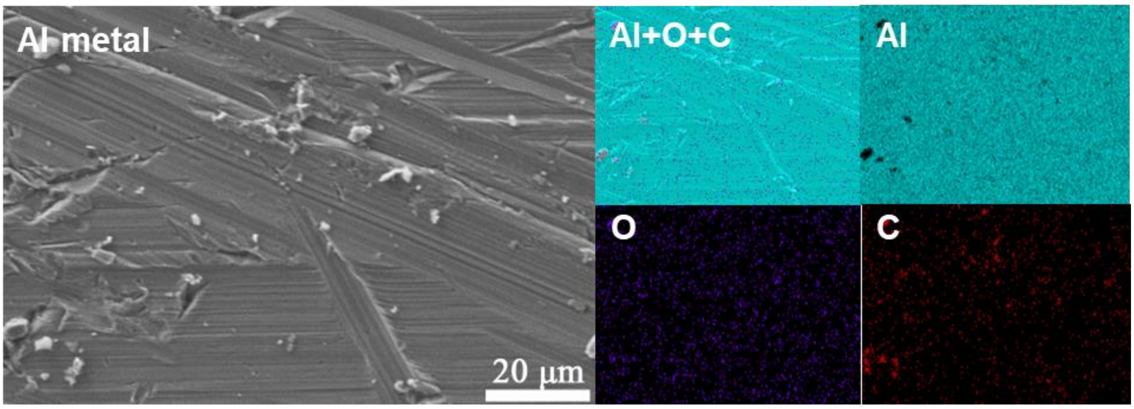

Figure S12. SEM image and EDX mapping of the initial Al anode.

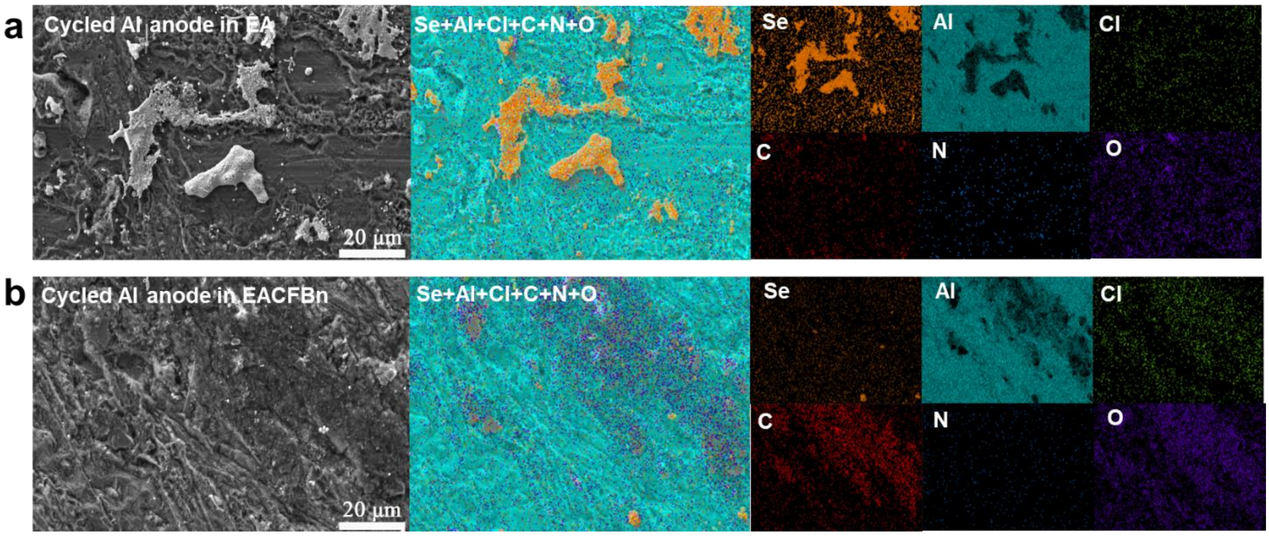

Figure S13. SEM image and corresponding EDX mapping of Al anodes cycled in (a) EA and (b) EACFBn.

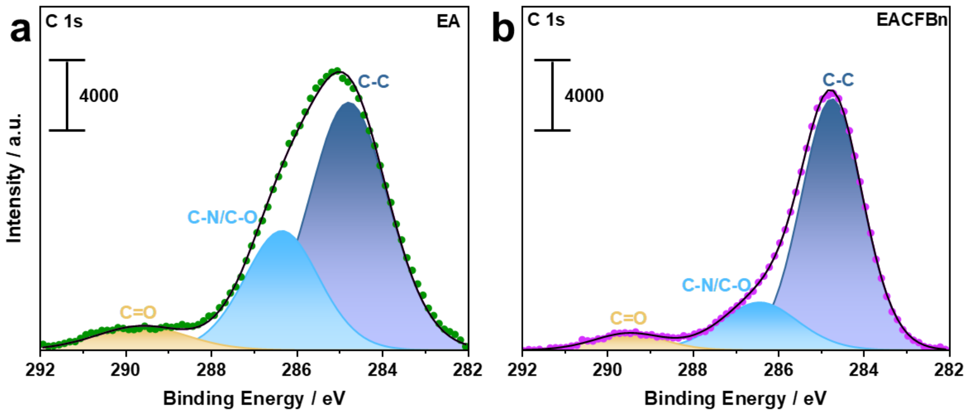

Figure S14. XPS detail spectra in the C 1s region of Al anode cycled in (a) EA and (b) EACFBn.
